# A Neuromorphic Digital Circuit for Neuronal Information Encoding Using Astrocytic Calcium Oscillations

**DOI:** 10.3389/fnins.2019.00998

**Published:** 2019-10-09

**Authors:** Farnaz Faramarzi, Fatemeh Azad, Mahmood Amiri, Bernabé Linares-Barranco

**Affiliations:** ^1^Department of Electronics, Amirkabir University of Technology, Tehran, Iran; ^2^Medical Technology Research Center, Kermanshah University of Medical Sciences, Kermanshah, Iran; ^3^Instituto de Microelectrónica de Sevilla (IMSE-CNM), CSIC and Univesity of Seville, Sevilla, Spain

**Keywords:** calcium modulation, astrocyte, information processing, neuromorphic circuit, FPGA

## Abstract

Neurophysiological observations are clarifying how astrocytes can actively participate in information processing and how they can encode information through frequency and amplitude modulation of intracellular Ca^2+^ signals. Consequently, hardware realization of astrocytes is important for developing the next generation of bio-inspired computing systems. In this paper, astrocytic calcium oscillations and neuronal firing dynamics are presented by De Pittà and IF (Integrated & Fire) models, respectively. Considering highly nonlinear equations of the astrocyte model, linear approximation and single constant multiplication (SCM) techniques are employed for efficient hardware execution while maintaining the dynamic of the original models. This low-cost hardware architecture for the astrocyte model is able to show the essential features of different types of Ca^2+^ modulation such as amplitude modulation (AM), frequency modulation (FM), or both modes (AFM). To show good agreement between the results of original models simulated in MATLAB and the proposed digital circuits executed on FPGA, quantitative, and qualitative analyses including phase plane are done. This new neuromorphic circuit of astrocyte is able to successfully demonstrate AM/FM/AFM calcium signaling in its real operation on FPGA and has applications in self-repairing systems. It also can be employed as a subsystem for linking biological cells to artificial neuronal networks using astrocytic calcium oscillations in future research.

## Introduction

Intracellular calcium (Ca^2+^) is a key second messenger in the living cells which controls various physiological processes by encoding information about external stimuli in amplitude or frequency of its oscillation (Woods et al., 1986; Kummer et al., 2000; Aguilera et al., 2019). Although many studies investigated the key role of intracellular Ca^2+^ oscillations, there are still many blind points. Indeed, the role of calcium oscillations in neural information encoding is still under investigation. The experimental observations support the fundamental role of astrocytes in forming Ca^2+^ oscillations. Researchers have shown that in response to a stimulation, astrocytes are able to release transmitters (called *gliotransmitters*) in a Ca^2+^-dependent manner and propagate intercellular Ca^2+^ waves over long distances. The discovery that calcium oscillations occur in astrocytes along with their ability to release gliotransmitters confirms that astrocytes actively participate in information processing in the brain (Rose and Karus, 2013; Oschmann et al., 2018). Given that astrocytes are not capable of creating action potentials, they respond to neuronal activity by increasing the cytosolic calcium level. Indeed, when an astrocyte is activated by its agonist (such as glutamate), a series of reactions is triggered, which leads to the production of IP_3_ (inositol 1, 4, 5-trisphosphate). Indeed, IP_3_ will trigger the calcium pathways through the IP_3_ receptor (IP_3_R), which releases calcium from the calcium store within the astrocytes. This calcium store is called the endoplasmic reticulum (ER). Consequently, astrocytes sense the neural transmission and respond by releasing different gliotransmitters, such as glutamate, Adenosine Triphosphate (ATP), and other neuroactive materials (Fields and Stevens-Graham, 2002; Min et al., 2012). In this way, astrocytes are active processing partners of neurons. Several experimental and theoretical studies are in progress to examine the computational power of neural-glial networks (Wade et al., 2011; Schafer et al., 2012; Liu et al., 2017). Additionally, there is also some evidence which suggests astrocytes participate in higher cognitive functions (Linne and Jalonen, 2014). Astrocytes may encode neural activity in different types of calcium responses (De Pittà et al., 2009; Dvorzhak et al., 2018). Various encoding patterns may explain how astrocytes can integrate synaptic transmissions and may represent different signaling mechanisms. It is also shown that different compartments of astrocytes (e.g., somata and endfeet) demonstrate specific types of calcium responses (Parpura, 2004; De Pittà et al., 2008).

In recent years, the function of neural mechanisms using digital and analog electronic systems are modeled (Indiveri et al., 2011; Frenkel et al., 2018; Yang et al., 2019). Many recent neuromorphic circuits have focused on single neuron (Wijekoon and Dudek, 2012), astrocyte (Ranjbar and Amiri, 2017), and neuron-astrocyte interactions (Soleimani et al., 2015; Karimi et al., 2018). Furthermore, other researchers have proposed a digital platform using a neural network and neuron-astrocyte interaction to investigate the self-repairing characteristics in FPGA (Liu et al., 2017; Karim et al., 2018). Johnson et al. used homeostasis in a spiking neural network to develop a fault-resilient robotic controller (Johnson et al., 2017). Recently they proposed a scalable FPGA-based hardware utilizing time multiplexing to design a self-repairing spiking astrocyte-neural network chip (Johnson et al., 2018).

However, a small number of the implemented circuits have been dealing with astrocytic Ca^2+^ signaling (Soleimani et al., 2015; Liu et al., 2017; Karimi et al., 2018), and none of them have proposed an analog or digital realization for information encoding based on astrocytic calcium oscillations. This in fact can be considered as a step forward in involvement of astrocytes in neuronal information processing from a hardware point of view. The recent introduced circuits (Soleimani et al., 2015; Ranjbar and Amiri, 2017; Karimi et al., 2018) have used the Postnov astrocyte model (Postnov et al., 2007), or its modified versions, which is a simplified, and non-dimensional model for the tripartite synapse. Nevertheless, this model and consequently its digital implementation do not consider the complex pathways of astrocyte calcium signaling, which should be taken into account for developing the next level of neuromorphic circuits. The Li and Rinzel (1994) or the Höfer et al. (2002) models are the main building blocks of the Ca^2+^-based excitability model of astrocytes (Manninen et al., 2018). De Pittà et al. (2009) extended the Li-Rinzel model to consider more intricate signaling. Specifically, they included calcium regulation by the IP_3_-dependent CICR (calcium-induced calcium-released) mechanism as well as IP_3_ dynamics resulting from PLC-mediated (phospholipase C) synthesis and degradation by IP_3_ 3-kinase and inositol polyphosphate 5-phosphatase. They showed that long-distance propagation of regenerative waves is closely related to the intracellular encoding of calcium responses. Frequency modulation encoding of calcium oscillations with pulsating dynamics induces regenerative waves that travel a long distance through gap junctions, while amplitude modulation encoding produces calcium waves that are constrained within a specific domain.

The main contribution of the current research is to design a neuromorphic circuit to encode information about external stimuli using different encoding approaches. This new digital circuit has the ability to switch among amplitude modulation (AM) of Ca^2+^ oscillations, frequency modulation (FM) of Ca^2+^ signaling, or combined AM and FM (AFM), which to the best of our knowledge have not been demonstrated in previous circuit realization. The proposed circuit can be used in the information processing section of the astrocyte-neuron network. Indeed, proposing low-cost and low-power hardware with the ability to code neuronal information has interesting applications in the self-repairing neural network, learning system and in linking biological neural networks with artificial neural systems. To this end, first, the nonlinear differential equations of the Ca^2+^ oscillations are simplified by a piecewise-linear approximation (PWL) method. Then, the obtained linear model is simulated in MATLAB and the results are compared with the original biophysical model. Next, a digital circuit is designed for the linear model and is then simulated in a Xilinx ISE (Integrated Synthesis Environment) simulation environment. Performing several experiments in different situations, it is shown that the new digital circuit follows the dynamical characteristics of the biophysical De Pittà model. Finally, the proposed digital astrocyte is run on the ZedBoard (Zynq Evaluation and Development kit) to get the real responses on the oscilloscope and validate the digital design. Changing the parameters of the digital circuit can switch the calcium oscillations among AM, FM, or AFM. All of these encoding approaches were successfully done in the real execution of the proposed circuit on the FPGA.

The rest of the paper is ordered as follows: in section Dynamic Models of Neuron and Astrocyte, the dynamic model of neuron-astrocyte crosstalk is explained. The proposed digital circuit is described in section Hardware Implementation. In section Results of simulations and hardware operation, the simulation and execution results are discussed. Finally, section conclusion describes the future directions and concludes the article.

## Dynamic Models of Neuron and Astrocyte

In this section, first, the Integrate & Fire (IF) neuron model is presented and then the biophysical model of astrocyte is explained.

### Neuron Mathematical Model

The IF model is one of the most common neuron models used in computational neuroscience, whose equation is as follows (Gerstner and Kistler, 2002):

(1) $$\begin{array}{l} \tau_{m}\frac{dV\left(t\right)}{dt}=-V\left(t\right)+R_{m}I_{syn} \end{array}$$

*R*_*m*_ is the membrane resistance, τ_*m*_ is the time constant, V is the membrane voltage and *I*_*syn*_ is the input current (from synapse). As the potential of the neuron membrane (V) reaches a threshold value (*V*_*th*_), V reset to 0. The IF neuron model parameters are shown in Table 1.

**Table 1:** Parameter values of the IF neuron model.

**Parameter**	**Value**	**Parameter**	**Value**
*I*_*syn*_	2	τ_*m*_	0.1
*R*_*m*_	2.5	*V*_*th*_	1

### Biophysical Model of Ca^2+^ Oscillations in Astrocyte

Astrocytes cannot produce action potentials; nevertheless, through bidirectional communication with neurons, they play a significant role in information processing (Haydon, 2001). It is currently obvious that astrocytes are active units which can regulate neuronal dynamics at the same or adjacent synapses. As a neuron fires, glutamate is released from the pre-synaptic neuron into the synaptic cleft, and it partially binds to the metabotropic receptors (mGluR) of the astrocytes (Porter and McCarthy, 1996). In fact, stimulation of astrocytes causes intracellular Ca^2+^ levels to increase due to the release of Ca^2+^ from Endoplasmic Reticulum (ER), mediated by IP_3_. IP_3_ is a glycoprotein that spontaneously induces calcium responses in astrocytes through IP_3_ receptors (IP_3_R) on the ER membrane. This leads to the release of calcium from the endoplasmic reticulum. Due to the presence of gap junction between astrocytes and thus forming astrocytic network, calcium waves can travel within the interconnected astrocyte network and allow the movement of IP_3_ into neighboring cells.

**Figure 1:**
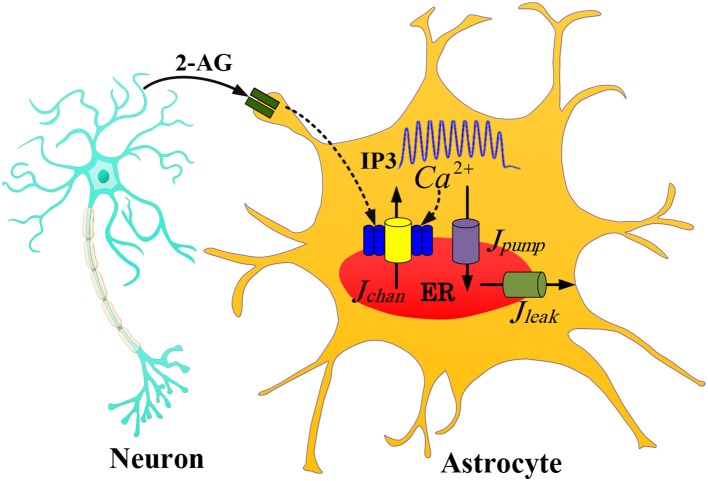
The communication between the IF neuron model and the astrocyte De Pittà model with one 2-AG signaling. The 2-AG signaling is indicated with the black line. As an action potential arrives at a presynaptic neuron, glutamate is released into the synaptic cleft which results in the release of 2-AG from a post-synaptic neuron (IF model). Then IP_3_ released into the astrocyte cytoplasm generates Ca^2+^ oscillations.

In this paper, we use the De Pittà model of astrocyte in the presence of indirect 2-AG (2-arachidonyl glycerol, a type of retrograde messengers) signaling. Figure 1 shows a neuron-astrocyte interaction with 2-AG signaling. We assume that when the IF neuron fires, 2-AG diffuses into the synaptic cleft. The quantity of propagated 2-AG is obtained from (2).

(2) $$\begin{array}{l} \frac{d\left(AG\right)}{dt}=-\frac{AG}{\tau_{AG}}+r_{Ag}\delta \left(t-t_{sp}\right) \end{array}$$

AG is the quantity of 2-AG, τ_*AG*_ is the decay rate of 2-AG, *r*_*AG*_ is the 2-AG generation rate and *t*_*sp*_ is the firing time of the IF neuron.

The IP_3_ is produced based on the gatekeeper model, when 2-AG binds to cannabinoid receptor 1 (CB1R) on the presynaptic terminal (Volman et al., 2007). The production of IP_3_ is influenced by the amount of propagated 2-AG. The production of IP_3_ within the astrocyte is modeled as:

(3) $$\frac{d\left(IP_{3}\right)}{dt}=\frac{IP_{3}^{*}-IP_{3}}{\tau_{ip3}}+r_{ip3}\left(AG\right)$$

*r*_*ip*3_ is the IP_3_ production rate, $IP_{3}^{*}$ is the baseline of IP_3_ (when the cell receives no input and it is in a steady state), τ_*ip*3_ is the IP_3_ decay rate.

The cytosolic calcium *Ca*^2+^ concentration is a function of the *Ca*^2+^ flux from the ER through the IP_3_ channels to the intracellular space (*J*_channel_), the leakage flux from the ER into the cytosol (*J*_Leak_), and pump-flux from the cytosol into the ER (*J*_Pump_). In the De Pittà model (De Pittà et al., 2008), the *Ca*^2+^ concentration in the intracellular space is explained by:

(4) $$\begin{array}{lll} \frac{d\left(Ca^{2+}\right)}{dt} & = & J_{chan}\left(Ca^{2+},h\;,IP3\right)+J_{leak}\left(Ca^{2+}\right)\\ \; & - & J_{pump}\left(Ca^{2+}\right) \end{array}$$

(5) $$\begin{array}{l} \frac{dh}{dt}=\frac{h_{\infty }-h}{\tau_{h}} \end{array}$$

where

(6) $$\begin{array}{l} h_{\infty }=\frac{Q_{2}}{Q_{2}+Ca^{2+}} \end{array}$$

(7) $$\begin{array}{l} \tau_{h}=\frac{1}{a_{2}\left(Q_{2}+Ca^{2+}\right)} \end{array}$$

(8) $$\begin{array}{l} Q_{2}=d_{2}\left(\frac{IP_{3}+d_{1}}{IP_{3}+d_{3}}\right) \end{array}$$

Where *h* is the fraction of activated IP_3_. The expressions for the fluxes are given by:

(9) $$\begin{array}{l} J_{pump}=v_{ER}\left(\frac{{\left(Ca^{2+}\right)}^{2}}{k_{ER}^{2}+{\left(Ca^{2+}\right)}^{2}}\right) \end{array}$$

(10) $$\begin{array}{l} J_{chan}=r_{c}m_{\infty }^{3}n_{\infty }^{3}h^{3}\left(c_{0}-\left(1+c_{1}\right)Ca^{2+}\right) \end{array}$$

(11) $$\begin{array}{l} J_{leak}=r_{L}\left(c_{0}-\left(1+c_{1}\right)Ca^{2+}\right) \end{array}$$

With

(12) $$\begin{array}{l} m_{\infty }=\frac{IP_{3}}{IP_{3}+d_{1}} \end{array}$$

(13) $$\begin{array}{l} n_{\infty }=\frac{Ca^{2+}}{Ca^{2+}+d_{5}} \end{array}$$

*r*_*C*_ denotes the maximal CICR rate, the total free *Ca*^2+^ cytosolic concentration is denoted by *c*_0_, *c*_1_ indicates the ER/cytoplasm volume ratio, the IP_3_ Induced Calcium Release (IICR), and CICR channels are represented by *m*_∞_ and *n*_∞_, respectively. *v*_*ER*_ is the maximum uptake rate for SERCA (Sarco-Endoplasmic-Reticulum *Ca*^2+^-ATPase) pump, *r*_*L*_ is the leakage rate of calcium and *k*_*ER*_ is the activation constant of the SERCA pump. The parameter values of models are given in Table 2 and taken from Gerstner and Naud, (2009).

**Table 2:** Parameter values of the De Pittà astrocyte model (Wade et al., 2011).

**Parameter values of astrocyte**		**Parameter values of astrocyte**	
**Parameters**	**Values**	**Parameters**	**Values**
τ_*AG*_	10 s	*AG*	0
*r*_*AG*_	0.018 μM s^−1^	*IP*_3_	0.16 μM
$IP_{3}^{*}$	0.16 μM	*Ca*^2+^	0.071006 μM
τ_*IP*3_	7 s	*h*	0.7791
*r*_*IP*3_	0.5 μM s^−1^	*m*_∞_	0
*a*_2_	0.2 μM	*n*_∞_	0
*d*_1_	0.13 μM	*J*_*chan*_	0
*d*_2_	1.049 μM	*J*_*leak*_	0
*d*_3_	0.9434 μM	*J*_*pump*_	0
*d*_5_	0.108 μM		
*c*_0_	2 μM		
*c*_1_	0.185 μM		
*r*_*L*_	0.11 s^−1^		
*v*_*ER*_	0.8 μM s^−1^		
*k*_*ER*_	0.1 μM		

The physiological role of astrocytic calcium oscillations in the encoding of synaptic information is still under investigation (De Pittà et al., 2009). Experimental observations suggest that the FM encoding is one of the main methods. In this way, synaptic activities are encoded in the frequency of astrocytic calcium oscillations (Parpura, 2004).

On the other hand, the possibility of AM encoding of synaptic transmission or AFM encoding has also been considered in recent theoretical and experimental works. Depending on the neuronal stimulation intensity, the amplitude of calcium oscillations in response to the external stimuli varies (De Pittà et al., 2009). Neurophysiological evidence suggests that astrocytes regulate synaptic information processing through calcium signaling. That is, the calcium oscillations characteristics such as amplitude and frequency are modulated by intrinsic properties of both neuronal inputs and the astrocyte's state. The De Pittà model for intracellular calcium signaling considers the diversity of the observed calcium dynamics when the biophysical parameters are varied. Recently it was shown that this model is able to illustrate encoding information about external stimuli by hiring different encoding modes. In this model, changes of biophysical parameters of the astrocyte may switch calcium signaling among AM, FM, or AFM (De Pittà et al., 2009).

## Hardware Implementation

**Figure 2:**
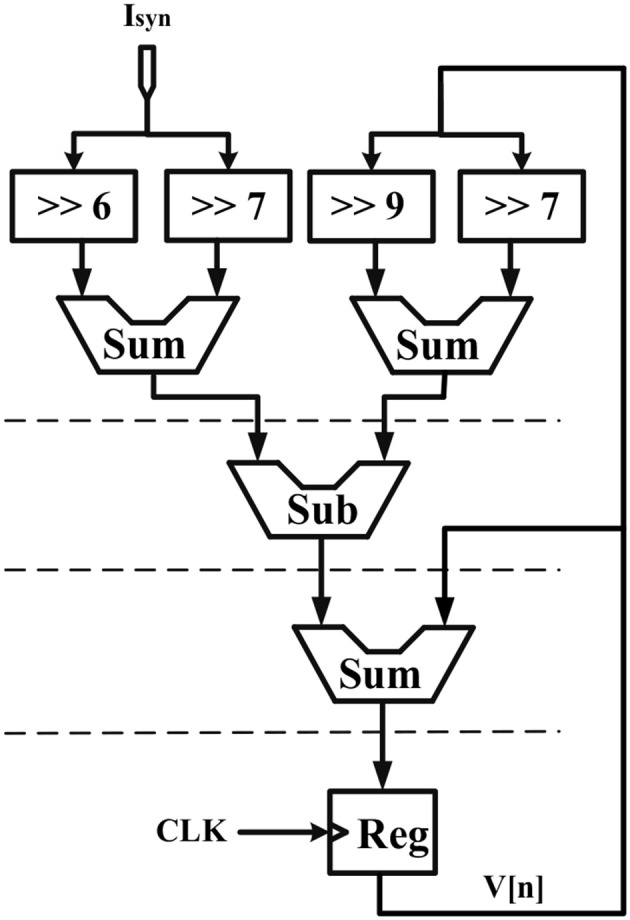
Scheduling diagram for the IF digital circuit. Action potential (*V*[*n*]) is produced.

**Figure 3:**
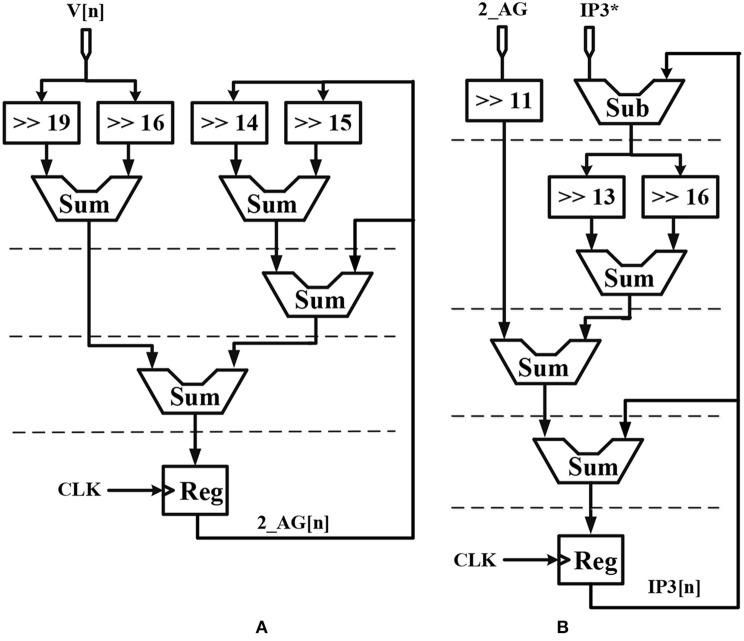
Scheduling diagram for **(A)** 2_*AG*[*n*] and **(B)** *IP*_3_[*n*] of the astrocyte.

**Figure 4:**
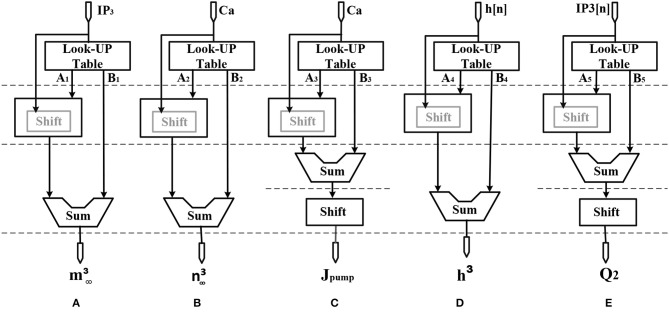
Scheduling diagram for **(A)** $m_{\infty }^{3}\left[n\right]$, **(B)** $n_{\infty }^{3}\left[n\right]$, **(C)** *J*_*pump*_[*n*], **(D)** *h*^3^[*n*], and **(E)** *Q*_2_[*n*].

**Figure 5:**
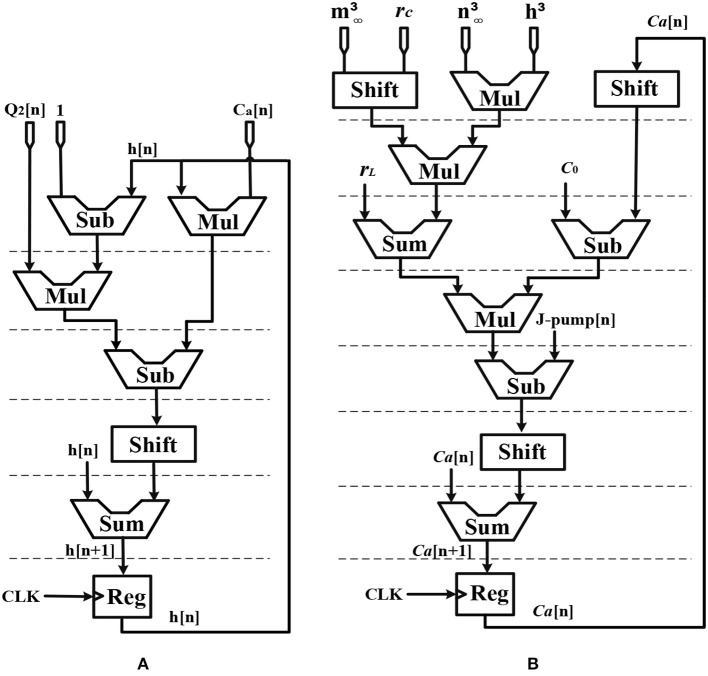
Scheduling diagram for **(A)** *h*[*n*] and **(B)** *Ca*^2+^[*n*].

Considering the main criteria from the hardware viewpoint, such as scaling up the designed circuit, reducing the implementation cost and keeping low power operation while obtaining results similar to the De Pittà model, we employ the piecewise-linear model to design efficient architecture to be run on the FPGA. Details of the hardware implementation are described in Appendix (Supplementary Material). Choosing the number of bits for individual variables is tied to the desired precision for realization, computational speed and resource utilization. In this research, a 39-bit fixed point (1 bit for sign, 4 bits for integer and 34 bits for fractional part) was used. Fixed-point computational units are typically faster and consume less hardware resources and power than floating-point engines. Bit-width of the parameters and variables are determined based on the two fundamental factors. These factors are the range of parameter variation and the spans of the shift operation. Moreover, considering the maximum shift operation (19 right-shift) and avoiding any overflow due to the shift operation while increasing computational accuracy, all variables and constants are restricted to the registers with 4 bits for the integer part and 34 bits for the fractional part. Figure 2 shows the scheduling diagrams for IF-neuron voltage (V[n]), and Figures 3–5 illustrate the proposed digital circuit for the astrocyte calcium oscillations, having the AM/FM/AFM properties. The neuron-astrocyte digital circuit was simulated and synthesized using VHSIC hardware description language (VHDL) and Xilinx ISE tools and was executed on the ZedBoard development kit. The maximum power dissipation of digital circuits was 78.45 mW. Tables 3, 4 show the summary of low and high levels of FPGA resource utilization for the digital circuits of astrocytes and neurons, respectively.

**Table 3:** Low-level device utilization summary for astrocyte and neuron.

	**Astrocyte synthesis report**			**Neuron synthesis report**		
**Slice logic utilization**	**Used**	**Available**	**Utilization**	**Used**	**Available**	**Utilization**
Number of slice LUTs	3,052	53,200	5%	180	53,200	0%
Number of bonded IOBs	3	125	2%	3	125	2%
Number of slice registers	262	106,400	0%	35	106,400	0%

**Table 4:** High-level device utilization summary for astrocyte and neuron.

	**Astrocyte**	**Neuron**
**Synthesis report**	**number**	**number**
39-bit Adders/Subtractors	16	4
Comparators	31	1
Multipliers	5	0
Multiplexers	111	1

### Results of Simulations and Hardware Operation

In this section, software simulation and hardware execution results are presented. Indeed, we investigate how hardware realization can produce the same results as MATLAB simulations.

**Figure 6:**
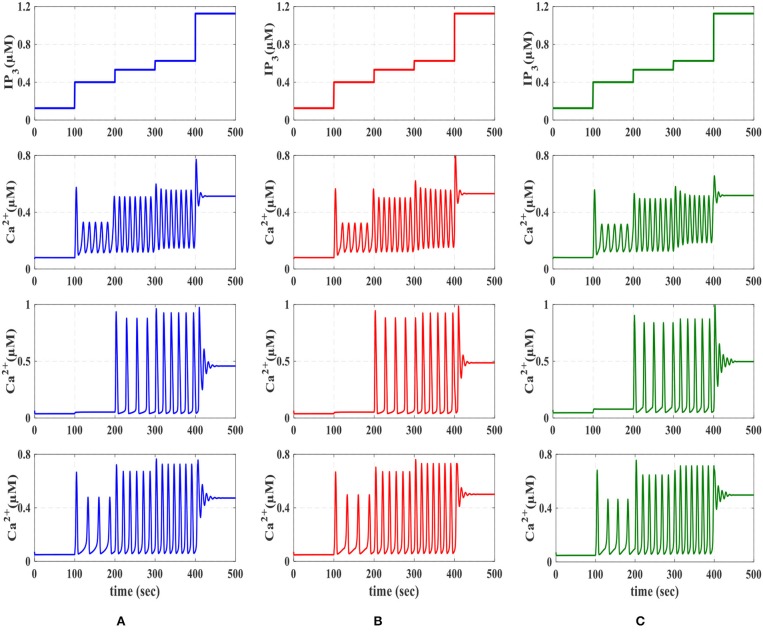
The first row is the IP_3_ stimulus signal with randomly incremented amplitudes. In this simulation with a time interval of 100s, the IP_3_ increment with *IP*_3_ = 0.125, 0.275, 0.13125, 0.09375, and 0.575 μM. The second row shows the results of AM modulation. The third row illustrates the results of FM modulation. The results of AFM modulation are presented in the last row. In each column, **(A)** depicts the results of biophysical model simulated in MATLAB, **(B)** shows the results of the linear model simulated in MATLAB, and **(C)** represents the ISE simulation of the proposed digital circuit.

Calcium dynamic is in its equilibrium state when the cytoplasmic calcium level is constant, $\frac{\text{d}\left({\text{C}\text{a}}^{2+}\right)}{\text{d}\text{t}}=0$, and the fraction of inactivated IP_3_R does not change, $\frac{\text{d}\left(h\right)}{\text{d}\text{t}}=0$. The stability of the equilibrium point depends on the IP_3_ level. At low IP_3_ values equivalent to basal condition or weak stimulation, the equilibrium point is stable, which in turn leads to the constant calcium. Such stability is then absent for higher values of IP_3_ concentrations, where Ca^2+^ oscillations increase in response to the external stimulus. Eventually, for higher values of IP_3_, the equilibrium becomes stable again. These observations can be summarized by noticing that the system dynamics change as the equilibrium changes from unstable to stable and vice versa (De Pittà et al., 2009). In Figure 6, the IP_3_ concentration is shown in the original De Pittà model (Figure 6A), the proposed piecewise-linear model (Figure 6B) and the proposed digital circuit (Figure 6C). In this simulation, we apply an incremental IP_3_ signal with a random level in each time interval of 100 s (the first row). As shown in the second row, when IP_3_ = 0.125 μM, the intracellular calcium level is constant. By increasing the IP_3_ content, the system loses its stability and the calcium amplitude elevates, thus IP_3_ information is encoded in the amplitudes of the intracellular calcium oscillations (AM). When IP_3_ = 1.2 μM or higher content, the calcium oscillations show a damping behavior and are steady in an overexcited calcium concentration. In the third row, for IP_3_ levels higher than 0.4 μM and less than 1.2 μM, the calcium dynamic loses its equilibrium and hence the information of IP_3_ excitation encoded in the frequency of intracellular calcium oscillations (FM).

**Figure 7:**
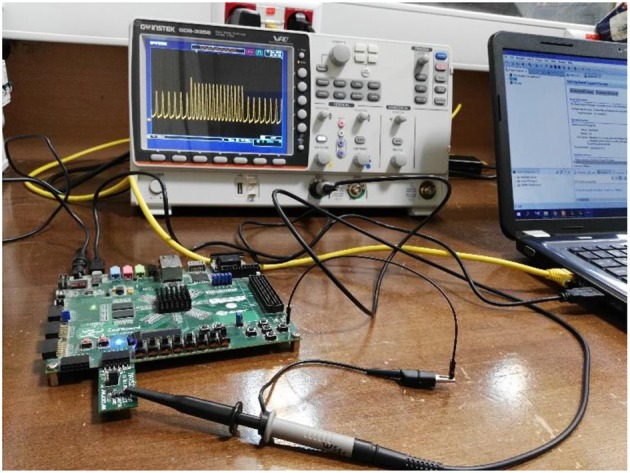
The physical setup for hardware testing of the proposed digital circuit for the De Pittà astrocyte model. In this case, the digital circuit is run on the ZedBoard, and the output signals after conversion to analog signals will be shown on the oscilloscope. For analog to digital conversion, a 10-bit ADC was used. However, a 16-bit DAC was utilized to convert the digital outputs of the ZedBoard to analog signals to be displayed on the oscilloscope.

**Figure 8:**
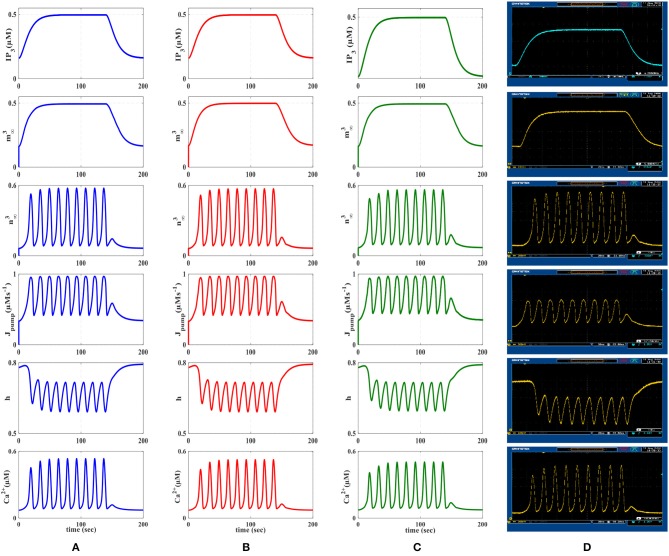
First row represents the *IP*_3_ signal that was produced as a result of receiving action potentials from the IF model. The other rows show the time response of the De Pittà astrocyte model ($m_{\infty }^{3}$, $n_{\infty }^{3}\;$, *J*_*pump*_, h, *Ca*^+2^). **(A)** MATLAB simulations of the biophysical De Pittà model. **(B)** The results of the linear model simulated in MATLAB. **(C)** VHDL simulations of the proposed digital circuit. **(D)** The photo of the oscilloscope screen when the designed digital circuit is running on the ZedBoard. Comparing each row, it is obvious that the general behavior of the acquired responses is preserved in real execution of the digital circuits.

**Figure 9:**
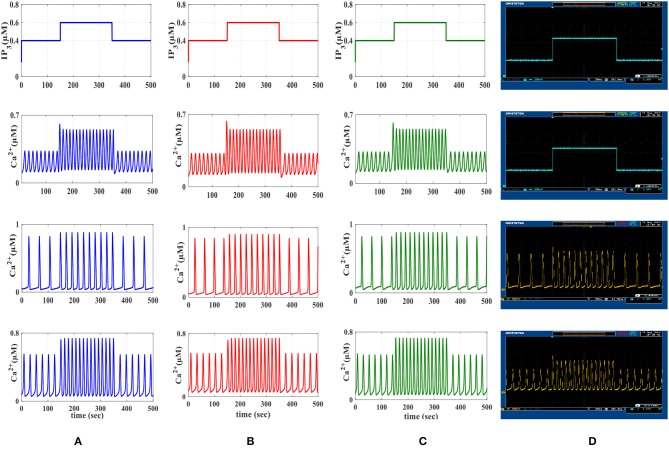
The first row is the IP3 stimulus signal with different amplitudes. In this experiment, the amplitude of the stimulus is set at 0.4 μM, then it is increased to 0.6 μM in the time interval 150–350 s and finally, it is reduced to its original value. The second row shows the results of AM mode with *r*_*L*_ = 0.014 and *k*_*ER*_ = 0.1. The third row shows the results of FM mode with *r*_*L*_ = 0.11 and *k*_*ER*_ = 0.064. The results of AFM mode with *r*_*L*_ = 0.07 and *k*_*ER*_ = 0.1 are shown in the last row. In each column, **(A)** depicts the results of biophysical astrocyte model simulated in MATLAB, **(B)** the results of linear model simulated in MATLAB, **(C)** represents the simulation of proposed digital circuits, and **(D)** illustrates the photo of the oscilloscope screen when the digital circuits are running on the ZedBoard.

Finally, the fourth row of Figure 6 depicts the stability behavior of the calcium signal for IP_3_ = 0.125 μM and 1.2 μM, and in this interval the information of external stimulation is encoded in both frequency and amplitude of intracellular calcium oscillations (AFM). Next, to identify the performance of the proposed astrocyte in hardware, the IF digital circuit is used to produce spike trains and thus to trigger the calcium oscillations within the astrocytes (releasing 2_AG to generate IP_3_). The produced IP_3_ causes *Ca*^2+^ variations to be initiated. The experimental setup to test the proposed digital circuit is shown in Figure 7. The digital circuits (Figures 2–5) are run on the ZedBoard. Figures 8C, 9C demonstrate the photo of the oscilloscope screen when FPGA executes the digital circuit. A 16-bit D/A converter (MAX5216PMB1 module) was used to convert the individual signal to an analog signal.

In Figure 8, the first row displays IP_3_, the second and third rows show $m_{\infty }^{3}\;$and $n_{\infty }^{3}$ functions, the fourth row is the dynamic of J_pump_ and the fifth and the last rows show the dynamic of *h* variable and the *Ca*^2+^ fluctuations, respectively. The first and second columns of Figure 8 represent the MATLAB simulations of the biophysical and linear models, the third column shows ISE simulations and the last column illustrates the oscilloscope screen for individual variables. Comparing different panels, it is apparent that VHDL simulations and digital circuit execution produce similar responses to the original biophysical model simulated in MATLAB, qualitatively.

Experimental observations propose that the frequency of intracellular calcium oscillations is likely to be the common way of synaptic activity encoding (Parpura, 2004). Increases in intensity or frequency of synaptic stimulation induce an equivalent increase in the frequency of calcium fluctuations. It should be pointed out that over the past years, it was shown that calcium signals in response to external stimuli may encode information through frequency modulation (FM) along with amplitude modulation (AM) (Berridge, 1997). While both types of dynamics have been seen separately, it is expected that they also coexist (Carmignoto, 2000). Nevertheless, the physiological bases for such coexistence are not yet understood well. In the AM mode, the peak value of calcium responses encodes the information on the level of *IP*_3_. It is directly linked to the strength of the stimulus affecting the cell. In the FM mode, variations in *IP*_3_ trigger calcium responses in which information is encoded in the inter-spike intervals. In the AFM case, both features contain information, which can be separately decoded by downstream effectors (Ono et al., 1999; John et al., 2001).

We will continue by performing further simulations to reveal the effectiveness of the proposed digital circuit in encoding external stimuli via complex intracellular calcium patterns either in the form of AM, FM, or AFM. In Figure 9, we apply the *IP*_3_ stimulus signal with different amplitudes. In this simulation, the initial value of the *IP*_3_ is set at 0.4 μM, at *t* = 150 s it is increased to 0.6 μM for 200 s and finally, at *t* = 350 s, it is reduced to its original value. Figure 9 shows the multimodal information encoding in the digital astrocyte. First, we apply the *IP*_3_ signal and, as seen in the second row, the amplitude of the calcium oscillations increases/decreases as *IP*_3_ increases/decreases, whereas their frequency is practically constant. Therefore, information about the level and amount of resealed *IP*_3_ is encoded in the amplitude but not in the oscillation frequency, so that the digital circuit shows the AM mode of information encoding. In the third row, information encoding in the FM mode can be seen easily. Indeed, as the level of *IP*_3_ changes, the amplitude of the Ca^2+^ oscillations is almost constant while the frequency increases accordingly. Finally, the fourth row shows the AFM information encoding mechanism by the digital astrocytes. In this case, any alteration at the level of *IP*_3_ not only changes the amplitude of Ca^2+^ oscillations but it also varies the frequency of oscillations as well. Noteworthily, the results of the MATLAB simulation, seen in Figure 9A, are in good agreement with the results obtained in ISE simulations in Figure 9B and with the real implementation of the digital circuit on the ZedBoard, Figure 9C. Indeed, Figure 9C shows the photo of the oscilloscope screen when the digital circuits are running on the ZedBoard.

**Figure 10:**
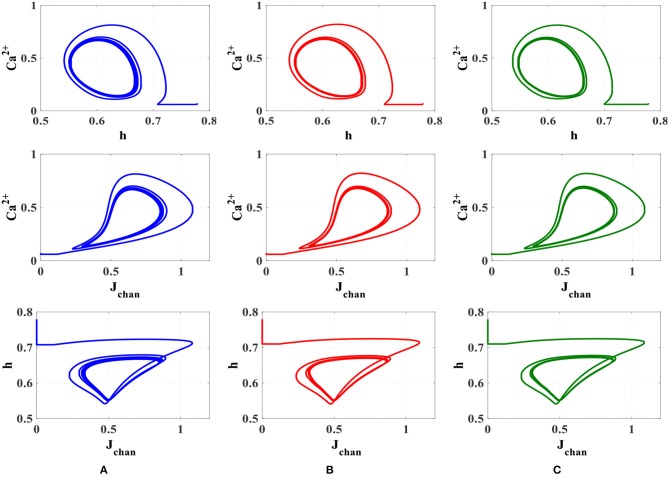
The phase plane analysis. **(A)** Original nonlinear model simulated in MATLAB, **(B)** the linear model simulated in MATLAB, **(C)** the proposed digital circuit.

To compare the dynamic behavior of the biophysical model and the proposed digital circuit, the phase planes (Ca^2+^ - h), (Ca^2+^ - J_chan_), and (h - J_chan_) are depicted in Figure 10. This test is commonly used in the study of dynamical systems to describe qualitative changes of the behavior of the system as one or more control parameters are altered (Amiri et al., 2011).

As can be seen, the dynamic behavior of the original model simulated in MATLAB, shown in Figure 10A, is similar to the qualitative behavior of trajectories in the linear model simulated in MATLAB (Figure 10B) and the proposed digital circuit (Figure 10C). In this way, the dynamic characteristics of the original model are maintained by the digital circuit, which was designed based on the linear model. Hence, not only the proposed neuromorphic circuit could maintain the performance and behavior of original model, but also by using the piecewise-linear model was low-cost hardware obtained.

To obtain a quantitative accuracy, we use the RMSE, which is calculated by (14). *Y*_*real*_ is the value obtained by a MATLAB simulation of the original model and *Y*_*digital*_ defines the value acquired by hardware running of the digital circuit. In addition, we calculate NRMSE, which is the normalized value of RMSE as computed in (15). The results of quantitative comparison between MATLAB simulation and the digital circuit in ISE are listed on Tables 5, 6, and very low values of RSME and NRSME show the reliability of the proposed digital circuit. Considering the fact that error accumulates through calculations, we calculated the error values in the 10th cycle to be sure that the errors converge to a nearly constant value.

(14) $$\begin{array}{l} RMSE\left(Y_{real},\;Y_{digital}\right)=\sqrt{\frac{\sum_{i=1}^{n}{\left(Y_{real}-Y_{digital}\right)}^{2}}{n}} \end{array}$$

(15) $$\begin{array}{l} NRMSE\left(Y_{real},\;Y_{digital}\right)=\frac{RMSE}{\;Y_{\max}-Y_{\min}\;} \end{array}$$

**Table 5:** The RMSE and NRMSE for the original model in MATLAB and digital realization in ISE (Figure 8).

**Function**	**RMSE**	**NRMSE**
V	0.0021	0.0020
AG	0.0043	0.004228
*IP*_3_	0.0196	0.05418
*J*_*pump*_	0.0561	0.0889
$m_{\infty }^{3}$	0.003	0.0091
$n_{\infty }^{3}$	0.0429	0.08626
*H*	0.0082	0.0418
*Ca*^2+^	0.0441	0.1018

**Table 6:** The RMSE and NRMSE for the original model in MATLAB and digital realization in ISE (Figures 6, 9).

**Function**	**Modulation**	**RMSE**	**NRMSE**
*Ca*^2+^		Figure 6			
	AM	0.1952	0.2782
	FM	0.2726	0.2910
	AFM	0.2184	0.3088
		Figure 9			
	AM	0.1739	0.3075
*Ca*^2+^	FM	0.2563	0.3083
	AFM	0.2304	0.3391

## Conclusion

Over the past decades, accumulating experimental and computational evidence expanded our knowledge about the key role of astrocytes in the brain and suggested that they are essential and active elements in neuronal information processing (Perea and Araque, 2010; Perea et al., 2014; Santello et al., 2019). Furthermore, new improvements in FPGA technology provide superior flexibility for algorithm exploration.

Although cellular calcium signaling was already used for realization of information processing (Heyde and Ruder, 2016), the use of astrocytic calcium oscillations in the neural information processing is less studied. The present study showed a new angle to analyze neuron–astrocyte crosstalk in hardware. Comparing with the other related digital implementations, to the best of our knowledge, this is the first neuromorphic circuit which realizes a more detailed model of astrocyte Ca^2+^ signaling. Indeed, in response to the external stimuli, the Ca^2+^ oscillations observed in the proposed digital circuit of astrocyte could encode information in the form of frequency modulation or amplitude modulation or both. Other previous hardware realizations (Soleimani et al., 2015; Ranjbar and Amiri, 2017; Karimi et al., 2018) have utilized a simple computational model of Ca^2+^ dynamics, and thus they are not able to show these information-encoding mechanisms. The results of running the proposed circuit on the FPGA illustrated acceptable performance with a very low error value between the proposed hardware and MATLAB simulations. Different types of information encoding including AM, FM, and AFM were successfully done in the real execution of the proposed circuit on the ZedBoard. Moreover, the proposed digital realization using PWL and SCM methods has consisted of simple arithmetic operations and has no important limitation.

The proposed hardware-based approach for encoding neural information through astrocytic calcium oscillations can be used in self-repairing neural networks (Liu et al., 2017) and spike-based learning mechanisms (Johnson et al., 2017, 2018) in spiking neural networks through astrocyte-neuron interactions. Future works will develop a network of these neuromorphic circuits to enhance the neuronal information processing/learning capabilities. Finally, the approach presented here may outline a new way to link neuronal/astrocyte cells to the hardware systems by connecting artificial and biological neural networks in future works.

## Data Availability Statement

The data supporting the conclusions of this manuscript will be made available by the authors to any qualified researcher upon request.

## Author Contributions

FF, FA, MA, and BL-B did conception and design, interpretation of results, drafting, and revising the article. FF and FA performed the experiments and acquired the data.

### Conflict of Interest

The authors declare that the research was conducted in the absence of any commercial or financial relationships that could be construed as a potential conflict of interest.
