# Supplementary material for: A Neuromorphic Digital Circuit for Neuronal Information Encoding Using Astrocytic Calcium Oscillations
Source: Front Neurosci. 2019 Oct 9;13:998. doi: 10.3389/fnins.2019.00998 (PMC6794439; doi:10.3389/fnins.2019.00998)
Supplement: Supplementary file 1 [file Data_Sheet_1.docx]

# Supplementary Material

## The Piecewise-Linear Model

In this section, for the De Pittà model of calcium oscillations within an astrocyte, a piecewise-linear approximation model (PWL) is offered. To stimulate the astrocyte, the IF neuron model is also implemented. Some essential criteria from the hardware viewpoint are considered: scaling up, reducing the realization cost and power and obtaining results similar to the biophysical model. Instead of using multipliers which influence area, latency and power consumption, other simple blocks such as shifters and adders are employed. Furthermore, the constant values of all equations are modified. This approach compensates the limited number of available multipliers on the chip, which makes it possible to implement a larger network on FPGA. The other main challenges are the presence of nonlinear terms which increase the implementation cost considerably. One way is to use a look-up table (LUT) to approximate nonlinear functions. Nevertheless, it needs several comparators and registers, which reduces the speed. To overcome this limitation, the piecewise linear method is used by trying to estimate the nonlinear relations with several line segments. This approach yields a linear system with similar dynamics to the original nonlinear model, which has been used widely in neuromorphic engineering, deep learning, artificial neural networks and biomimetic circuits to achieve a simple and low-cost hardware (Ehlers, 2017; Pillai et al, 2019; Yang et al, 2018; Morales et al, 2015).

In the first step, the dynamical equation of the IF neuron (Eq.1) and the AG and IP_3_ equations (Eqs.2, 3) are considered. Since they are linear, they can be easily discretized as follows:

| $V\left[ n+1 \right]=V\left[ n \right]+dt.{\tau_{m}}^{-1}( -V\left[ n \right]+R_{m}.I_{syn})$ | (16) |
| --- | --- |
| $AG\left[ n+1 \right]=AG[n]+dt.( -{\tau_{\mathrm{AG}}}^{-1}.AG\left[ n \right]+ r_{AG}.V\left[ n \right])$ | (17) |
| ${IP}_{3}\left[ n+1 \right]={IP}_{3}\left[ n \right]+dt.( {\tau_{ip3}}^{-1} .\left( {{IP}_{3}}^{*}-{IP}_{3}\left[ n \right] \right)+r_{ip3}.AG[n])$ | (18) |

Next, the Single Constant Multiplication (SCM) procedure is applied to adjust the constants in Eq. (16) -(18) so they can be rewritten based on the power of 2. This supports executing constant multiplication simply by logical shift and add/subtract operations. The modified equations are:

| $V\left[ n+1 \right]=V\left[ n \right]-(2^{-9}+ 2^{-7}).V\left[ n \right]+(2^{-6}+ 2^{-7})I_{syn}$ | (19) |
| --- | --- |
| $AG\left[ n+1 \right]=AG\left[ n \right]-(2^{-14}+ 2^{-15}).AG\left[ n \right]+ (2^{-16}+ 2^{-19}).V\left[ n \right])$ | (20) |
| ${IP}_{3}\left[ n+1 \right]={IP}_{3}\left[ n \right]+(2^{-13}+2^{-16}) .\left( {{IP}_{3}}^{*}-{IP}_{3}\left[ n \right] \right)+2^{-11}.AG[n]$ | (21) |

Next, we substitute $J_{chan}$ and $J_{leak}$ from (10, 11) into ${Ca}^{2+}$. Then, following Liu et al. (2018) and Yang et al. (2015) and using the Euler method, the continuous time equations are discretized to get discrete ${Ca}^{2+}[n]$ (see eq.(20)) by substituting (6,7) into (5) to describe *h*[n] as shown in (23). In these equations, *dt* is 0.001, the discretizing step size.

| ${Ca}^{2+}\left[ n+1 \right]={Ca}^{2+}\left[ n \right]+dt.( (r_{L}+r_{C} \left( m_{\infty}\left[ n \right] \right)^{3} \left( n_{\infty}\left[ n \right] \right)^{3} {h\left[ n \right]}^{3} )\times(c_{0}-\left( 1+c_{1} \right){Ca}^{2+}\left[ n \right])- J_{pump}\left[ n \right] )$ | (22) |
| --- | --- |
| $h\left[ n+1 \right]=h\left[ n \right]+dt.(a_{2}Q_{2}\left[ n \right]\left( 1-h\left[ n \right] \right)-a_{2}h\left[ n \right]\left( {Ca}^{2+}\left[ n \right] \right))$ | (23) |

For the ${Ca}^{2+}$ equation, individual parts such as $m_{\infty}^{3}$, $n_{\infty}^{3}$, $h^{3}$ and $J_{pump}$ should be calculated. We start with ${m_{\infty}}^{3}$, which consists of two nonlinear terms ($\frac{X}{X+c}$,$X^{3}$). These nonlinear terms increase the implementation cost considerably. Using the linear approximation method, the nonlinear relations are estimated by several line segments. In this case, nonlinear parts are substituted with some first order functions. To determine the coefficients of linear functions while obtaining minimum approximation error, an exhaustive search algorithm is utilized. The algorithm started by selecting two arbitrary points on the target curve by the user. In other words, three intervals on the nonlinear curve are nominated. Then, the least square method is applied to find the best fitted linear equation in each interval. We used root-mean-square error (RMSE) to compute the error between piecewise-linear model and nonlinear curve. If the obtained RMSE was lower than a specific threshold (in this work, 0.04), the search algorithm will be terminated and the coefficients of the linear equations are determined, otherwise the positions of the initial points are changed to find the linear equation with the lower RMSE value. The aforementioned process has been repeated from 2 to 7 initial points on the target curves. This threshold has been chosen experimentally in a way that the proposed digital circuit not only mimics the behavior of the biophysical model of astrocyte but avoids the use of extra logic sources. Moreover, we considered the threshold value which also has been reported in (Yang et al., 2018). Considering the circuit complexity and achieving appropriate accuracy, $m_{\infty}^{3}$ is simplified with 6 lines. The approximated $m_{\infty}^{3}$ equation is as follows:

| $m_{\infty}^{3}=A_{1}.{IP}_{3}[n]+B_{1}$ | (24) |
| --- | --- |

where $A_{1}$ and $B_{1}$ are the line parameters as listed in Table 7, and the approximated $m_{\infty}^{3}$ is shown in Figure 12. The process starts with applying the input ${IP}_{3}$ to the corresponding approximation unit, and based on the input region and Table 7, the appropriate $A_{1}$ and $B_{1}$ are selected. They will be updated after each iteration by the next state. Similar to $m_{\infty}^{3}$computation, the $n_{\infty}^{3}$ function is approximated by 7 lines, as shown in Figure 13(a). The line parameters are chosen from Table 8. The third nonlinear function in (20) is the $h^{3}$, which can be written as follow:

| ${h[n]}^{3}=A_{3}.h[n]+B_{3}$ | (25) |
| --- | --- |

Because the h is a positive function, we approximate the $h^{3}$ function only for positive values of input. As shown in Figure 13(b), $h^{3}$ is also approximated with 7 lines. The values of $A_{3}$ and $B_{3}$ with respect to the input range are listed in Table 9. The last nonlinear term for implementing the ${Ca}^{2+}$ equation is $J_{pump}$, which can be approximated as follows:

| $J_{pump}[n]=v_{ER}\times(A_{4}{Ca}^{2+}\left[ n \right]+B_{4})$ | (26) |
| --- | --- |

and is shown in Figure 14, with the parameter values listed in Table 10. Finally, to calculate $h\left[ n+1 \right]$, the nonlinear term of $Q_{2}[n]$ should be linearized. As depicted in Figure 15, this term is approximated with 5-line segments, for which the coefficients are listed in Table 11. The approximated $Q_{2}$ is as follows:

| $Q_{2}[n]=d_{2}\times(A_{5}{IP}_{3}[n]+B_{5})$ | (27) |
| --- | --- |

It should be pointed out that in the above equations, constant values are rounded to the nearest 2’s complement values. This helps to run the digital circuit with fewer shifters and adders.

**FIGURE 12.** 6-line segments approximation for $m_{\infty}^{3}$

|  |  |
| --- | --- |
| (a) | (b) |

**FIGURE 13**. (a) 7-line segments approximation for $n_{\infty}^{3}$. (b) 7-line segments approximation for $h^{3}$

**TABLE 7 |** The parameter values of individual lines for the 6-line segments approximation of $m_{\infty}^{3}=A_{1}\mathrm{IP}_{3}+B_{1}$

| B_1_ | A_1_ | Region of IP_3_ ($\mu M$) |
| --- | --- | --- |
| 0 | 0.18 | [ 0 , 0.03182 ) |
| -0.035 | 1.28 | [ 0.03182 , 0.26 ) |
| 0.0291 | 1.0333 | [ 0.26 , 0.3358 ) |
| 0.1107 | 0.7904 | [ 0.3358 , 0.4474 ) |
| 0.2037 | 0.5825 | [ 0.4474 , 0.6554 ) |
| 0.3835 | 0.3082 | [ 0.6554 , 1.3 ) |

**FIGURE 14**. 6-line segments approximation for $\frac{J_{pump}}{V_{ER}}$

**FIGURE 15**. 5-line segments approximation for $\frac{Q_{2}}{d_{2}}$

**TABLE 8 |** The parameter values of individual lines for 7-line segments approximation of $n_{\infty}^{3}=A_{2}\mathrm{Ca}^{2+}+B_{2}$

| B_2_ | A_2_ | Region of Ca^2+^ ($\mu M$) |
| --- | --- | --- |
| 0 | 0.55 | [ 0 , 0.0489 ) |
| -0.0556 | 1.6870 | [ 0.0489 , 0.1827 ) |
| 0.0059 | 1.3504 | [ 0.1827 , 0.2556 ) |
| 0.0822 | 1.0563 | [ 0.2556 , 0.3533 ) |
| 0.19608 | 0.73396 | [ 0.3533 , 0.4868 ) |
| 0.3072 | 0.5057 | [ 0.4868 , 0.6659 ) |
| 0.4480 | 0.2943 | [ 0.6659 , 1 ) |

**TABLE 9 |** The parameter values of individual lines for 7-line segments approximation of $h^{3}=A_{3} . h$ + $B_{3}$

| B_3_ | A_3_ | Region of h |
| --- | --- | --- |
| 0 | 0.0089 | [ 0 , 0.1385 ) |
| -0.0176 | 0.1360 | [ 0.1385 , 0.2906 ) |
| -0.0847 | 0.3669 | [ 0.2906 , 0.4097 ) |
| -0.2119 | 0.6774 | [ 0.4097 , 0.552 ) |
| -0.4846 | 1.1715 | [ 0.552 , 0.694 ) |
| -0.8899 | 1.7555 | [ 0.694 , 0.832 ) |
| - 1.4539 | 2.4334 | [ 0.832 , 1 ) |

**TABLE 10 |** The parameter values of individual lines for the 6-line segments approximation of $\left( \frac{\left( {Ca}^{2+} \right)^{2}}{k_{ER}^{2}+\left( {Ca}^{2+} \right)^{2}} \right)=A_{4}{Ca}^{2+}+B_{4}$

| B_4_ | A_4_ | Region of Ca^2+^ ($\mu M$) |
| --- | --- | --- |
| - 0.1 | 6.0940 | [ 0 , 0.09634 ) |
| 0.0954 | 4.0658 | [ 0.09634 , 0.146 ) |
| 0.3762 | 2.1425 | [ 0.146 , 0.2036 ) |
| 0.6127 | 0.9809 | [ 0.2036 , 0.3015 ) |
| 0.8194 | 0.2952 | [ 0.3015 , 0.4895 ) |
| 0.9344 | 0.0602 | [ 0.4895 , 1 ) |

**TABLE 11 |** The parameter values of individual lines for the 6-line segments approximation of

$$\left( \frac{\mathrm{IP}_{3} +d_{1}}{\mathrm{IP}_{3} + d_{3}} \right)=A_{5}\mathrm{IP}_{3}+B_{5}$$

| B_5_ | A_5_ | Region of IP_3_($\mu M$) |
| --- | --- | --- |
| 0.1389 | 0.8345 | [ 0 , 0.091 ) |
| 0.1521 | 0.6891 | [ 0.091 , 0.21 ) |
| 0.1874 | 0.521 | [ 0.21 , 0.4 ) |
| 0.2440 | 0.3794 | [ 0.4 , 0.655 ) |
| 0.3209 | 0.2620 | [ 0.655 , 1.3 ) |
